# Characterization of the Wild Trees and Shrubs in the Fergana Valley: Diversity and Distribution, Threats

**DOI:** 10.1002/ece3.73632

**Published:** 2026-05-08

**Authors:** Nazokat Daminova, Xian‐Han Huang, Hushbaht R. Hoshimov, Dilmurod Makhmudjanov, Farkhod I. Karimov, Hee‐Young Gil, Komiljon Sh. Tojibaev, Hyeok Jae Choi

**Affiliations:** ^1^ The Laboratory of Flora of Uzbekistan, The Academy of Sciences of the Republic of Uzbekistan Institute of Botany Tashkent Uzbekistan; ^2^ State Key Laboratory of Plant Diversity and Specialty Crops Kunming Institute of Botany, The Chinese Academy of Sciences Kunming China; ^3^ Department of Biology Namangan State University Namangan Uzbekistan; ^4^ Forest Biodiversity Research Division, Korea National Arboretum Pocheon Republic of Korea; ^5^ Department of Biology and Chemistry Changwon National University Changwon Republic of Korea

**Keywords:** altitudinal zonation, biodiversity conservation, Chorkesar, dendroflora, Eastern Alay, floristic composition, threats

## Abstract

This study aimed to investigate the floristic characteristics, biogeographic distribution, and altitudinal zonation of native trees and shrubs (over 50 cm in height) in the flora of the Fergana Valley (Uzbekistan). Field surveys at 32 sites recorded 165 species from 60 genera and 32 families, including 5 (3.03%) subendemic taxa listed in the national Red Book. Among the families, 14 are represented by a single species each, 10 families by 2–9 species each, and 5 families by 10–15 species each, while the family Rosaceae is represented by 42 species. 26 genera were each represented by a single species, while 34 genera were each represented by 2–10 species, and the genus *Rosa* was represented by 11 species. Among the life forms represented in the dendroflora, phanerophytes are the most dominant (135 taxa), followed by chamaephytes (30 taxa). The results of the floristic analysis revealed a clear predominance of temperate taxa across all hierarchical levels. At the species level, Central Asian and Temperate Asian elements together accounted for 87.89% of the flora, indicating the distinctly temperate nature of the regional dendroflora and underscoring the crucial role of endemic species in maintaining biodiversity. All taxa inhabit six natural habitat types. Botanical–geographic analysis of the highest species richness was found in the Chorkesar (62.42%) and Eastern Alay (52.72%) regions, while the East–Fergana region contained the lowest (11.51%). According to the results of altitudinal distribution, mountain areas harbored the highest tree and shrub diversity (112 taxa), while the high‐altitude pasture zone is the poorest (7 taxa). The highest diversity along horizontal and vertical gradients was observed in the Rosaceae family (300–3100 m), with peak species richness recorded between 1400 and 2100 m. These findings provide an important scientific reference for identifying regional biodiversity hotspots, conserving rare species, supporting future floristic and biogeographic research.

## Graphical abstract

The content of this paper is significant because it presents the first comprehensive assessment of the dendroflora of the Fergana Valley, contributes to understanding species richness across biogeographic regions, and places the results in the context of Central Asian flora and global biodiversity studies.

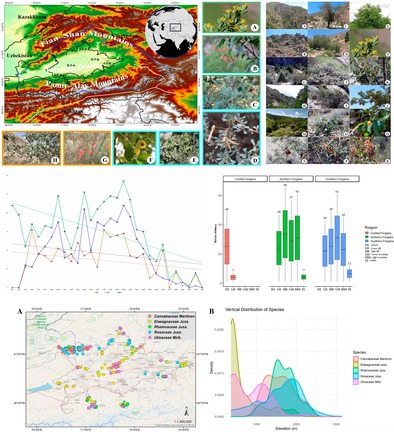

## 1 Introduction

A number of studies have so far been carried out on the inventory of tree and shrub biodiversity in the world flora, including “The chronology of trees and shrubs in South‐west Asia and adjacent regions” (Browicz 1982; Mehrabian et al. 2020; Zohary 1971); the Tertiary flora of the Euro‐Siberian region in Southwest (SW) Asia (Profile 2017; Thalen 1979; Zakirov 1947); patterns of distribution in woody zones of high diversity worldwide (Du et al. 2015; Mehrabian et al. 2020; Takhtajan 1986; Van Heezik et al. 2014; Vivero et al. 2005); and conservation status (Eastwood et al. 2009; FAO 1986; Vivero et al. 2005). Newton and Oldfield (2008) reviewed the latest advances in the Red List of the world's tree species. In addition, Beech et al. (2017), Bedair et al. (2020), Liu et al. (2024), Manvelidze et al. (2010), Médail et al. (2019), Meddour et al. (2021), Rejmánek and Richardson (2013), Roma‐Marzio et al. (2016), Sterling and Orr (2014), and Song et al. (2021) have made significant contributions to the study of tree and shrub flora. The Red List of trees in Central Asia (Eastwood et al. 2009), the Red List of maples (Gibbs and Chen 2009), the conservation of fruit trees in Central Asia, the global list of invasive alien trees and shrubs (Roma‐Marzio et al. 2016), and the distribution patterns of tropical timber species have been published (Van Heezik et al. 2014). At this point, it is worth emphasizing that the tree and shrub taxa occurring in the flora of Central Asia have not yet been fully studied or inventoried.

Central Asia is considered a global biodiversity hotspot (Myers et al. 2000; Profile 2017). This region comprises Kazakhstan, Kyrgyzstan, Tajikistan, Turkmenistan, and Uzbekistan states, and, due to its geographical structure, contains numerous small subregions and extensive altitudinal belts surrounded by some of the world's highest mountain ranges, such as the Tian–Shan and the Pamir–Alay. The flora of the Tian–Shan and Pamir–Alay mountains is extremely rich but remains insufficiently studied (Tojibaev et al. 2022). In particular, the tree and shrub taxa occurring in this region have not yet been fully inventoried. According to the latest data reported by Eastwood et al. (2009), approximately 500–600 species of trees and shrubs are estimated to occur in the flora of Central Asia. However, the critical analysis of the lists of tree and shrub taxa presented in classical works on the floras of Kazakhstan, Kyrgyzstan, Turkmenistan, Tajikistan, and Uzbekistan showed that the dendroflora of Central Asia consists of more than 665 species belonging to 115 genera and 44 families. It is possible that these numbers may be higher or lower due to the integration of results from floristic studies conducted over the past 30 years. In addition, the trees and shrubs and flora of Uzbekistan, which is part of Central Asia—particularly the dendroflora of the Fergana Valley—has not yet been studied. However, in the first publication of the flora of Uzbekistan (published between 1941 and 1962), about 365 dendroflora species belonging to 72 genera and 37 families were reported (Kudryashev 1941; Vvedensky 1953–1962). The study area, the Fergana Valley, encompasses nearly 45.20% of the country's dendroflora species, and accounts for about 25% of the woody plants of the Central Asian region. The Angren Plateau of the Fergana Valley (70,000 ha, of which 45,000 ha fall within the study area), the upper reaches of the Chodak and Chorkesar rivers (53,000 ha), the Pap foothills (24,000 ha), Karatag (4000 ha), Ungor Tepa (2000 ha), the Chartak foothills (2000 ha), the Akkum sands (11,000 ha), the upper Syrdarya basin (4000 ha), the Teshiktosh foothills (27,000 ha), the Chilustun and Kirtashtau mountains (6000 ha), Shakhimardan (4000 ha), and Sokh (20,000 ha) have been recognized as Key Biodiversity Areas (KBAs) of Central Asian mountains by leading specialists in the field, as documented in a monograph (Profile 2017). These areas are characterized by high botanical value and particular significance. Moreover, they harbor a high proportion of rare, endemic, and threatened plant species. Therefore, conserving the diversity of tree and shrub taxa present in this area is of great importance not only at the local but also at the regional scale.

Fergana Valley (Uzbekistan territory) land (about 22,000 km^2^) consists of four geographical districts according to its natural geographical location (Dekhkonov et al. 2021; Tojibaev et al. 2017), namely, Western Tian–Shan (about 3800 km^2^), Fergana (about 1618.9 km^2^), Fergana–Alay (about 5070.62 km^2^), and Central Fergana (about 11,344.9 km^2^) districts. The geological history and flora and endemism of Tian–Shan and Pamir–Alay mountains surrounding the valley, as well as the structure and vegetation cover of the Central Fergana exclave separating these mountains, are divided into two provinces (Tojibaev et al. 2016, 2017, 2020). These provinces include the following: (1) Mountainous Central Asian province, which includes the following: the Kurama ridge of Western Tian–Shan, the southern branches of the Chatkal range, and the lower parts of the Poshshaota basin; Pamir–Alay range, which includes mountain and sub‐mountain areas of the Alay range; (2) The lowlands of Central Fergana, which are considered a special exclave of the Turan desert province, separating the Tian–Shan and Pamir–Alay ranges, include the Karakalpak, Yazyavan, and Kayrakum deserts, the Syrdarya river, and its three old gorges.

Fergana Valley has been recognized by a number of researchers as the most densely populated area in Central Asia, and the area of natural landscapes is extremely limited (Daminova et al. 2023, 2024; Hoshimov 2023; Karimov 2016; Tojibaev 2002). The region is characterized by a continental climate, distinguished by low humidity and predominantly arid conditions (Daminova et al. 2023, 2024; Daminova and Tojibaev 2024; Hoshimov 2023; Karimov 2016; Tojibaev 2002; Tojibaev et al. 2022, 2023, 2025). As a result, the desert region, which makes up 50% of the Fergana Valley, is not conducive to the establishment of true forests with large trees and shrubs. The widely distributed plants in this region have deep root systems, which allow them to adapt to nutrient‐poor soils; the plants are resistant to high radiation levels and drought, and salt, and the presence of thorns and small leaves characterizes the semi‐arid environment and exhibits a number of morphophysiological characteristics. In particular, a large amount of NaCl, Na_2_SO_4_, and NaCO_3_ salts accumulates in the stems and small fleshy leaves (Crisp and Lange 1976). Many trees and shrubs are of great economic value in arid regions (Bedair et al. 2020; Crisp and Lange 1976). They also play an important role in soil protection and stabilization against wind or water movement, are a source of fodder for animals and fuel for local people, and have medicinal and potential industrial values (Bedair et al. 2020; Crisp and Lange 1976; Thalen 1979).

About 10% of the area occupied by the Fergana Valley is mountainous (Daminova and Tojibaev 2024). On the one hand, this situation confirms the lack of natural landscapes in the valley area; on the other hand, the sparse juniper forests in the mountain region, xerophytic trees and shrubs adapted to arid climatic conditions, and broad‐leaved forests in some areas with sufficient moisture contain high biodiversity, and the most plant species represent one of the rich habitats. These forests provide very important ecosystem services, such as regulating the climate of the valley, cleaning the atmosphere, producing biomass, mitigating the negative consequences of the global climate change process, creating unique habitats for forest species, and providing renewable raw materials for industry and the national economy. Additionally, the trees and shrubs present in these forests play a significant role in mitigating floods and preventing floods from mountain valley rivers, as well as dust storms caused by strong winds.

Plants, especially trees and shrubs, which are one of the most important components of the natural ecosystems of the Fergana Valley, are affected by human activity (firstly, the annual increase in the number of the population and the increase in the demand for housing; secondly, the increase in the rate of economic growth and the level of urbanization; thirdly, high‐speed exploitation of natural landscapes for planting agricultural crops (extermination) and overgrazing of livestock, unfortunately in danger of extinction as a result of related disturbances (Daminova and Tojibaev 2024).

Over the past 50 years, some natural landscapes have been destroyed at a high level of anthropogenic factors (Tojibaev et al. 2022, 2023, 2025). As a result, in scientific literature, Bondarenko (1955), Arifkhanova (1967), Pratov (1970), Khalkuziev (1971), Tojibaev (2002) conducted floristic and geobotanical studies of numerous species (trees: *Betula tianschanica*, *Crataegus chlorocarpa* ( *C. altaica* , *C. korolkowii*), *Malus sievesii*, *Hedlundia persica* (*Sorbus persica*), *Populus pruinose*, *P. euphratica*, *Salix iliensis*, *S. turanica* , *Sorbus tianschanica* , *Rhamnus cathartica* , *Haloxylon ammodendron* (*Haloxylon aphyllum*); shrubs: *Astragalus spryginii*, *Convolvulus spinifer*, *Convolvulus fruticosus*, *Lonicera bracteolaris*, *Lonicera webbiana* (*Lonicera karelinii*, *Lonicera heterophylla* ), *Ribes meyeri* ) populations have sharply declined, and some species disappeared. In particular, *Sorbaria olgae*, collected by O.A. Fedchenko in 1871 from the Alay Mountain Range of the Pamir–Alay [Kокaнcкоe xaнcтво. Ущeльe бл. Шaшмapдaн. 7.VII.1871. O.A. Φeдчeнко! (sub *S. sorbifolia* )], was first described as a new species for science by Yu.D. Tsingerling (G. Zinserling) in 1926 in Volume VI of Botanicheskie Materialy. By 1970, populations of this species had declined due to strong anthropogenic pressures (Daminova and Tojibaev 2024). Khalkuziev (1971) reported that this species was absent from the Shakhimardan River basin flora. The species included in the Red Books of Kyrgyzstan (2006) and Uzbekistan (1998, 2006) in order to protect and preserve the species (Davletkeldiev 2006; Pratov 1998, 2006). Later, K.Sh. Tojibaev (2002–2010), N.E. Daminova (2020–2024), and others on the flora of the Alay Range (around the village of Shakhimardan) did not record *S. olgae* . The scarcity of natural landscapes in the study area, the continuous reduction in forest‐covered areas, and the high level of influence of other anthropogenic factors require special attention to endemic, rare, and endangered species, including representatives of the dendroflora of the Fergana Valley.

The flora of the valley includes relatively few trees and shrubs. Large‐scale botanical research conducted in this area dates back to the 50–80 s of the last century. However, until now, the composition of the dendroflora of the Fergana Valley has not been studied. Therefore, it is of great ecological importance to make a complete inventory of the species composition of trees and shrubs distributed in the Fergana Valley region with arid climatic conditions. This article has the following goals: (1) to present the first complete list of local dendroflora growing in the natural landscapes of the Fergana Valley, to determine the presence of rare and endemic or near‐endemic species; (2) description of taxonomic and chorological diversity, life forms; (3) geographical distribution, horizontal and vertical gradient distribution patterns, conservation status of rare and endemic species, analysis of threatened taxa.

## 2 Material and Methods

### 2.1 Data Collection and Sources

The information sources for compiling data on the tree and shrub flora of Fergana Valley were various and heterogeneous. The bibliographic research started with the “Materials for flora of Fergana,” by Fedchenko and Fedchenko (1902), “Vegetation of the Ferghana Valley,” by Arifkhanova (1967), which served as an important starting point for our inventory. This flora remains up to now the main source of information for any floristic study in Fergana Valley. Tree species data were also gathered from other relevant primary sources, such as the “Vegetation of the Namangan region and its economic importance” (Bondarenko 1955), “The haze of the Ferghana Valley” (Pratov 1970), “Flora and vegetation of the Shakhimardan River basin” (Khalkuziev 1971), and Vegetation cover and meadows of the Chodaksoy basin (Tojibaev 2002). However, several knowledge gaps exist at different levels (e.g., distribution of trees and shrubs, species in need of protection, etc.). We filled these gaps by conducting numerous field studies. For example, main habitats, coordinates, uses and threats for taxa were recorded through visiting different locations.

In 2020–2023, in order to collect trees and shrubs of the Fergana Valley, field research was carried out in 32 areas in many places of the Fergana Valley. Botanical–geographical regions where the expedition was conducted are as follows: Arashan, Kurama, Chorkesar, Southern Chatkal, Garbiy–Alay, Eastern Alay, Kayrakum–Yazyavan, Eastern Alay (Table A1). From each location, specimens of plant taxa were collected from different habitats, and herbarium collections representing the recorded taxa were also collected whenever possible. Field studies were carried out using floristic route and semi‐stationary methods, and a total of more than 5100 herbarium specimens were collected during the research. Additional data were obtained from major herbaria such as TASH, LE, and MW. To collect more information about the listed plants, to check some identifications, and to obtain additional information, the websites given in Table A2 were consulted.

### 2.2 Floristic Analyses

The species which were not collected from the field were examined from herbarium sheets deposited in LE, MW, and TASH. The list of families (Table 1) is arranged alphabetically according to the APG IV (The Angiosperm Phylogeny Group and A New Classification of Gymnosperms) system (Byng et al. 2016; Christenhusz et al. 2011). In addition, the checklist of the Tian–Shan mountain system flora by Sennikov and Tojibayev was used. Identification of plant specimens was carried out depending on the previous mentioned literature. In addition, re, the information portals Plantarium.ru (www.theplantlist.org) and iNaturalist (www.inaturalist.org), The Plant List (http://www.theplantlist.org), International Plant Name Index (IPNI (http://www.ipni.org)), Global Plant Science (JSTOR) (http://plants.jstore.org), Kew world checklist of different plant families (http://wcsp.science.kew.org), Global Biodiversity Information Facility (GBIF (http://www.gbif.org/occurence)) were also consulted. Some identifications were revised in TASH based on reference materials. The taxonomic richness of the flora is expressed in number of taxa in a given area (Tojibaev et al. 2016, 2017), and provides information on the number of genera and species per family, and species by genus (Sennikov and Tojibaev 2021). This is both the simplest and most easily interpreted measure of taxonomic diversity (Whittaker et al. 2001).

**TABLE 1:** Taxonomic diversity of the trees and shrubs in the Fergana Valley flora.

Family	Genus		Species		Rare	
	Ac	Re	Ac	Re	Ac	Re
Gymnosperms						
Cupressaceae	1	1.66	3	1.81	—	—
Ephedraceae	1	1.66	2	1.21	—	—
Angiosperms (Eudicots)						
Rosaceae	11	18.33	42	25.45	—	—
Fabaceae	4	6.66	15	9.09	—	—
Amaranthaceae	9	15.00	14	8.48	—	—
Salicaceae	2	3.33	14	8.48	—	—
Polygonaceae	2	3.33	11	6.66	2	1.21
Plumbaginaceae	1	1.66	10	6.06	1	0.60
Tamaricaceae	2	3.33	9	5.45	1	0.60
Caprifoliaceae	2	3.33	9	5.45	—	—
Berberidaceae	1	1.66	4	2.42	—	—
Lamiaceae	3	5.00	4	2.42	—	—
Solanaceae	1	1.66	3	1.81	—	—
Ranunculaceae	1	1.66	3	1.81	—	—
Elaeagnaceae	2	3.33	2	1.21	—	—
Zygophyllaceae	1	1.66	2	1.21	—	—
Convolvulaceae	1	1.66	2	1.21	—	—
Rhamnaceae	1	1.66	2	1.21	—	—
Cistaceae	1	1.66	1	0.60	—	—
Nitrariaceae	1	1.66	1	0.60	—	—
Betulaceae	1	1.66	1	0.60	—	—
Sapindaceae	1	1.66	1	0.60	—	—
Oleaceae	1	1.66	1	0.60	—	—
Cannabaceae	1	1.66	1	0.60	—	—
Anacardiaceae	1	1.66	1	0.60	—	—
Juglandaceae	1	1.66	1	0.60	—	—
Asteraceae	1	1.66	1	0.60	—	—
Thymelaeaceae	1	1.66	1	0.60	1	0.60
Grossulariacea	1	1.66	1	0.60	—	—
Phyllanthaceae	1	1.66	1	0.60	—	—
Celastraceae	1	1.66	1	0.60	—	—
Ulmaceae	1	1.66	1	0.60	—	—
Total	60	100	165	100	5	3.1

Abbreviations: Ac, actual number; Re, relative number (%).

### 2.3 Life Form and Floristic Element

Life forms of the recorded taxa were assessed using the system of Raunkier (1934). In addition, when performing the analysis of floristic elements, the distribution type at the family, genus, and species levels was determined based on the classification of Wu (1991), Wu et al. (2003) and Huang et al. (2025).

### 2.4 Analysis of Distribution of Taxa Across Botanical–Geographic Regions and Elevation Gradients

The distribution of species within the dendroflora across botanical–geographic regions was carried out according to the botanical–geographic zoning scheme of Uzbekistan proposed by Tojibaev et al. (2016). The altitudinal distribution of species was determined using the classification for the Central Asian region proposed by Acad. Zakirov (1947). To determine the relationship between elevation and species richness across botanical–geographical regions, georeferenced (coordinate‐based) data were collected based on field studies and herbarium records from the study area. An elevation value (in meters) was assigned to each observation using a Digital Elevation Model (DEM). Species richness was calculated as the number of plant species within each elevation range. To determine the linear relationship between elevation and species richness, Ronald A. Fisher's–*R* ^2^–coefficient of determination (used to assess the correlation between two variables) and PERMANOVA analysis (*p*‐value coefficient) were applied.

#### 2.4.1 Statistical Analysis

To determine the linear relationship between elevation and species richness, a simple linear regression analysis was performed. The model was defined in R (using the lm() function). The model is expressed in the following form:

$$R^{2}=1-\frac{ss_{\mathrm{res}}}{ss_{\mathrm{tot}}}$$

where

*R* ^2^ = 1—The model explains all of the variation, SS_res_—residual sum of squares, SS_tot_—total sum of squares.

##### 2.4.1.1 Model Evaluation: *R* ^2^ and *p*‐Value

The following statistical indicators were used to assess the model's effectiveness:

Coefficient of determination (*R* ^2^): The *R* ^2^ value indicates the proportion of variation in species richness that can be explained by elevation. The value ranges from 0 to 1. For example, if *R* ^2^ = 0.60, this means that 60% of the variation in species richness can be explained by elevation.

##### 2.4.1.2 *p*‐Value (PERMANOVA Analysis)

The *p*‐value evaluates whether there is a significant relationship between elevation and species richness. If the *p*‐value ranges from < 0.01 to < 0.05, the relationship is considered statistically significant and reliable. If the *p*‐value is greater than 0.05, the relationship is not statistically significant, meaning that elevation does not have a measurable effect on species richness. This analysis was conducted based on the following formula.

$$p=\frac{1+\sum_{i=1}^{n_{\text{perm}}}I\left(F_{\text{perm},i}\geq F_{\mathrm{obs}}\right)}{1+n_{\text{perm}}}$$

where

$F_{\mathrm{obs}}$—the actual observed pseudo‐*F* value, $F_{\text{perm},i}$—*F* values resulting from permutations, $n_{\text{perm}}$—overall number of permutations (usually 999 or 9999).

## 3 Results

### 3.1 Floristic Analysis

A total of 165 tree and shrub species (including 163 native and 2 invasive taxa), representing 60 genera and 32 families, were documented during this 4‐year study. Most of the taxonomic units belong to Angiospermae, and they lead the dendroflora of the research area with 160 species (96.96%) belonging to 58 genera and 30 families. The flora consists of 5 species ( *Ephedra equisetina* , *Ephedra intermedia*, *Juniperus semiglobosa*, *Juniperus seravschanica* , and *Juniperus pseudosabina* ) belonging to gymnosperms and two genera of Cupressaceae and Ephedraceae. These species represent the principal structural element of the mountainous vegetation within the investigated area.

The spectrum of leading families in dendroflora is represented by Rosaceae (42 spp., 25.45%), Fabaceae (15 spp., 9.09%), Amaranthaceae (14 spp., 8.48%), Salicaceae (14 spp., 8.48%), Polygonaceae (11 spp., 6.66%), and Plumbaginaceae (10 spp., 8.48%, 6.00%), Caprifoliaceae, and Tamaricaceae (9 species each, 5.45%). These families include 124 species and cover 75% of all tree and shrub species in the dendroflora of the Fergana Valley (Table 1). Between 4 and 2 species were observed in the families Berberidaceae and Lamiaceae (4 species each), Cupressaceae, Solanaceae, Ranunculaceae (3 species each), Elaeagnaceae, Ephedraceae, Zygophyllaceae, Convolvulaceae, and Rhamnaceae (2 species each). It was determined that the remaining families will participate with one species each.

The most represented genera are *Rosa* (11 spp., 6.66%), *Acantholimon*, *Prunus*, *Salix* (10 species each, 6.06%), *Astragalus* (9 spp., 5.45%) and *Lonicera*, *Tamarix* (8 species each, 4.84%). Also in the top 10 are the genera *Calligonum*, *Crataegus* (6 species each, 3.63%), *Cotoneaster*, and *Atraphaxis* (5 species each, 3.03%) (Table A3). This means that 53% of the tree flora is grouped within these six genera. Then, 49 genera encompass between four and one taxa, while 3.03% of all genera (6 species) are monospecific in the tree flora of Fergana Valley.

The conducted research revealed the absence of any species endemic to the Fergana Valley of Uzbekistan. The category of subendemics (species whose distribution range is mainly confined to a certain region, or partly extends into neighboring areas, are considered subendemic species) includes five species considered rare for the flora of the study area: *Acantholimon margaritae*, *Calligonum calcareum*, *C. elegans* , *Lonicera paradoxa*, *Restella albertii*. Species whose distribution range is mainly confined to a certain region, or partly extends into neighboring areas, are considered subendemic species.

### 3.2 Life Forms

The life form determination of the recorded species indicated that phanerophytes (135 taxa = 81.82% of the total recorded taxa) are the most represented life form, followed by suffruticose chamaephytes (30 taxa = 18.18%) (Appendix A). Class of phanerophytes based on height: nano‐phanerophytes (shrubs, 50 cm–2 m, 84 taxa = 50.90%), micro‐phanerophytes (small trees, 2–8 m, 43 taxa = 26.03%), and meso‐phanerophytes (medium‐sized trees, 8–30 m, 8 taxa = 4.84%).

The location of the Fergana Valley between the Tian–Shan and Pamir–Alay mountains, combined with its continental climate, significantly influences the distribution and development of dendroflora species in the area. Climate factors, such as humidity and annual rainfall, affect various life forms under environmental influences, impacting their growth. Notably, species belonging to the megaphanerophyte group, which can reach heights over 30 m under optimal humidity conditions, are absent here. A contributing factor to the scarcity of mesophanerophyte and microphanerophyte tree species in the dendroflora is that a large part of the study area consists of desert and hilly regions, with unique natural and climatic conditions in these areas.

### 3.3 Floristic Elements

The 165 taxa identified for the dendroflora of the Fergana Valley were assigned to different floristic elements based on Wu (1991), Wu et al. (2003), and Huang et al. (2025) (Table 2). The results showed that the taxa listed in the dendritic flora can be classified into 7 distribution types. At the family level, the widespread element (Type 1) constituted the largest proportion, comprising 18 families and accounting for 56.25% of the total, followed by the temperate element (Types 8 and 12) with 11 families (34.38%), among which the distribution type of North Temperate (Type 8) was the most represented with 9 families. In contrast, the tropical element was exclusively represented by three pantropical families (Type 2): Anacardiaceae, Phyllanthaceae, and Sapindaceae. At the genus level, temperate elements remained dominant, comprising 50 genera (83.33% of the total), with the Type 8 again being the richest (23 genera), while the tropical element included only three pan‐tropical genera (5%). Notably, at the species level, only temperate elements were present. The Central Asian element (Type 13) was the most species‐rich (97 species, 58.79%), followed by the distribution type of Temperate Asian (Type 11) with 48 species; collectively, these two types accounted for 145 species, representing 87.89% of the total species richness.

**TABLE 2:** Distribution types of tree and shrub taxa.

Distribution type	Family number	Percentage (%)	Genus number	Percentage (%)	Species number	Percentage (%)
1. Widespread	18	56.25	7	11.67	—	—
2. Pantropic	3	9.38	3	5	—	—
8. North Temperate	9	28.13	23	38.33	1	0.60
10. Old World Temperate	—	—	7	11.67	14	8.48
11. Temperate Asia	—	—	2	3.33	48	29.1
12. Mediterranea and West to Central Asia	2	6.25	15	25	5	3.03
13. Central Asia	—	—	3	5	97	58.79
Total	32	100	60	100	165	100

*Note:* — represents zero.

### 3.4 Habitat Types

The most widespread natural habitat is forests (i.e., evergreen, semi‐evergreen, broad‐leaved, and mixed forests, with 62 taxa (the forests located in the Chokesar and Eastern Alay botanical–geographical regions)), followed by sand dunes, sandy soils, and saline soils (i.e., sandy soil, desert plains, sandy plains, wet and soft saline soils, saline sandy soils, takyr, gypsum soils, and saline loess soils, with 38 taxa) (Table 3). For example, species belonging to the families Rosaceae (*Rosa*, *Prunus*, *Crataegus*, *Cotoneaster*, *Spiraea*), Fabaceae (*Astragalus*, *Caragana*), Caprifoliaceae (*Lonicera*), and Cupressaceae (*Juniperus*) form mountain and steppe forests, which are relatively widespread in the northern and southern parts of the Fergana Valley. Additionally, species from the families Amaranthaceae (*Haloxylon*, *Xylosalsola*, *Suaeda*) and Polygonaceae (*Calligonum*) are mainly distributed in desert regions, and play a significant role in forming the desert forests of Kayrakum, Yazyavan, and Karakalpak located in the central part of the Fergana Valley. The remarkable diversity of tree taxa is found in rocky habitats (rocky mountain ridges and cliff coasts) that provide favorable conditions (shelters) and are remote from anthropogenic factors, encompassing 29 taxa (Table 3). Twenty‐seven taxa are found in water basin shores and marshy areas (in freshwater, brackish, or saline streams), such as species from the families Tamaricaceae (*Tamarix*) and Salicaceae (*Salix*); these species form riparian forests, typically located along ephemeral rivers (valleys). Other habitats host fewer taxa: pastures and grazing lands (7 taxa) and synanthropic habitats (2 taxa).

**TABLE 3:** Relative proportion of the six habitat types in which the tree flora occurs in Fergana Valley.

Habitat types	Number of taxa	Proportion (%)
Forest habitat	62	37.58
Pastures and grasslands	7	4.24
Rocky habitat	29	17.58
Sandy habitat	38	23.03
Synanthropic habitat	2	1.21
Water basin shores and marshy habitat	27	16.36

### 3.5 Distribution by Botanical and Geographical Regions

**FIGURE 1:**
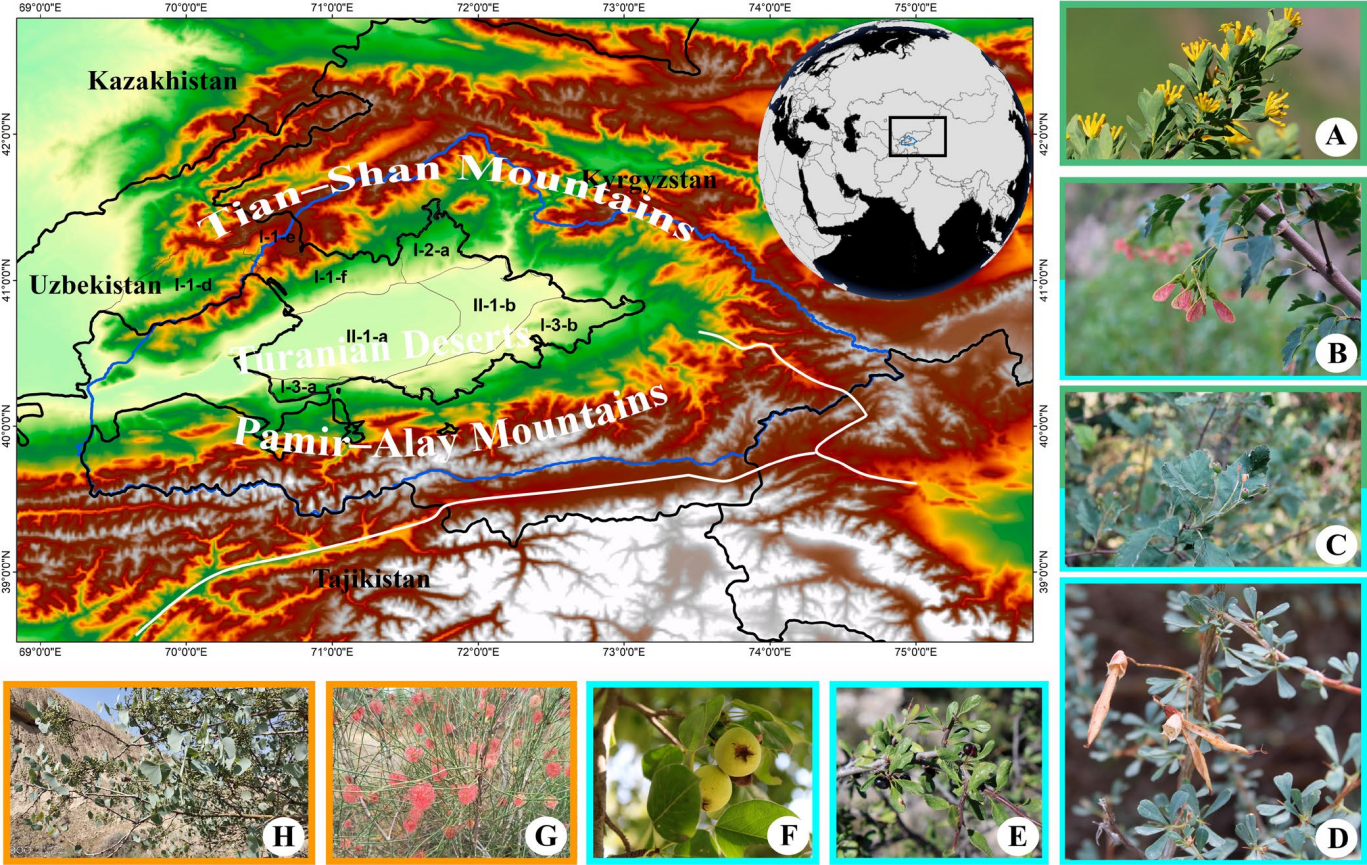
Fergana Valley within the territory of Uzbekistan. (A) Restella albertii, (B) *Acer tataricum* subsp. semenovii, (C) Hedlundia persica, (D) Caragana alaica, (E) Rhamnus songorica, (F) Pyrus korshinskyi Tian–Shan and Pamir–Alay mountains species; (G) *Calligonum caputmedusae* , (H) Populus pruinosa Turan area species. (A–F, H) central Asia types; (G), temperate Asia types: I Mountain Central Asian Province: I‐1‐c Arashan, I‐1‐d Kurama, I‐1‐e Charkesar, I‐2‐a South–Chatkal, I‐3‐a Western–Alay, I‐3‐b Eastern–Alay. II Turan Province: II‐1‐a Kayrakum–Yazyavan, II‐1‐b East–Fergana.

The distribution of the dendroflora of the Fergana Valley by botanical–geographical regions (BGR) was carried out according to the scheme of botanical–geographical regionalization of Uzbekistan proposed by Tojibaev et al. (2016, 2017). A high proportion of the taxonomic diversity within the dendroflora (86.66%) was attributed to the Mountain Central Asian province. A total of 143 identified species were recorded in this province. The Mountain Central Asian province is further subdivided into the Western Tian–Shan, Fergana, and Fergana–Alay districts (Figure 1).

In terms of species richness, the Western Tian–Shan district holds the leading position, encompassing 109 species of trees and shrubs, which accounts for 66.06% of the total. The Fergana–Alay district ranks next, with 106 species (64.24%) of trees and shrubs. The main reasons for the high richness of tree and shrub species in the Western Tian–Shan and Fergana–Alay districts are as follows: (1) A significant part of the Kurama mountain range is located within the Western Tian–Shan district, with its highest elevation reaching 3800 m. Moreover, the majority of the area covered by this range falls within the territory of the Fergana Valley. (2) A small part of the Alay mountain range is included in the Fergana–Alay district, with its highest elevation reaching 3200 m. The Alay range is considered one of the areas within the Fergana Valley with a high level of biodiversity. The Fergana district hosts a relatively lower number of species, with its species diversity accounting for 35.15% (58 spp.) of the total dendroflora (Table A4). This situation is explained by the relatively smaller total area of the district compared to the other two districts, as well as the lower elevation range, which is less favorable for the distribution of tree and shrub species.

The Turan Province is represented by a small proportion of species within the dendroflora composition. In this part of the study area, 53 species (32.12%) were recorded. The Kayrakum–Yazyavan and Eastern Fergana districts of the Central–Fergana region are included within this province. The main portion of the taxonomic richness was recorded in the Kayrakum–Yazyavan district, which accounted for 27.27% (45 species) of the total (Table A4).

#### 3.5.1 Chorkesar BGR (Figure 1, I‐1‐e)

**FIGURE 2:**
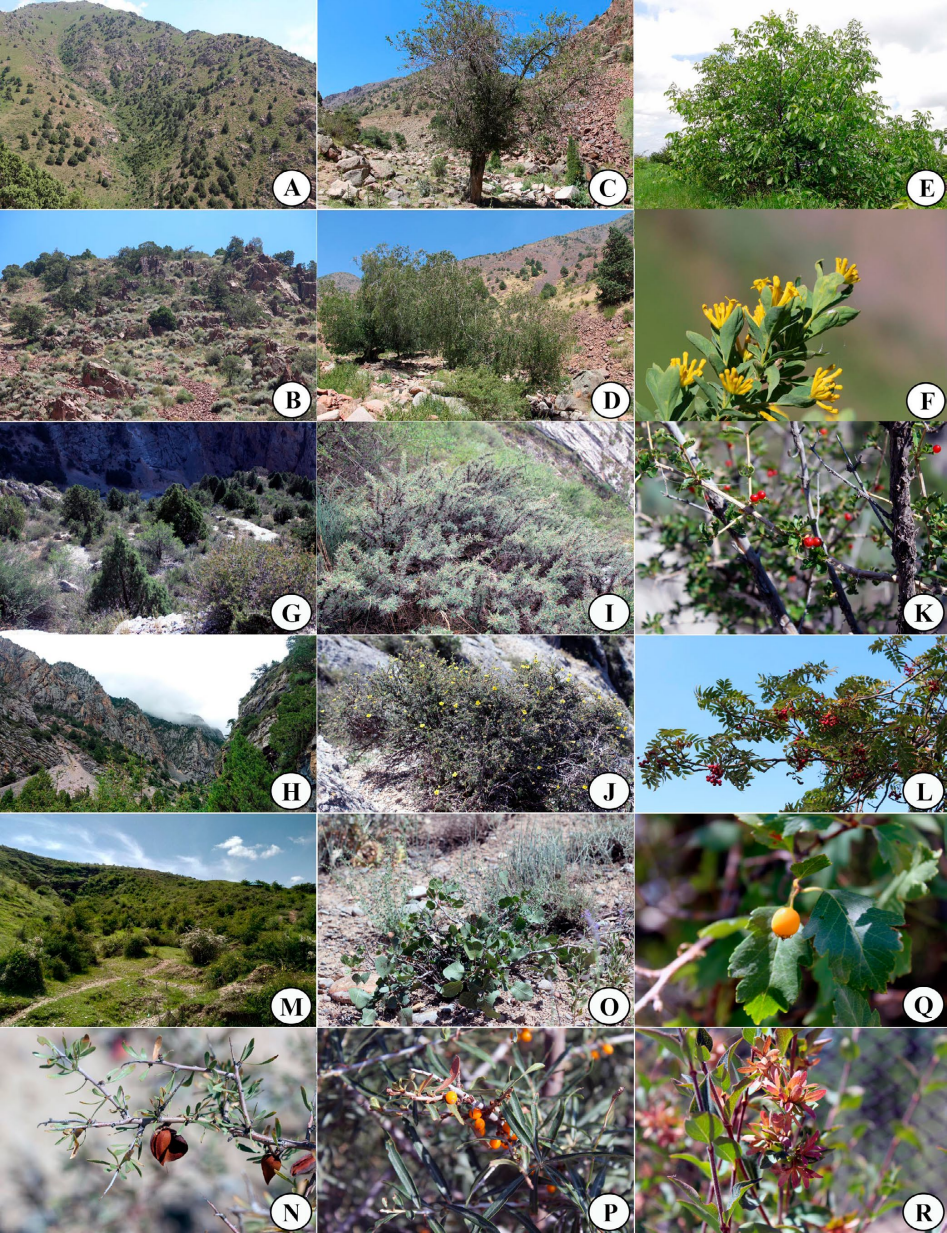
Chorkesar region: (A, B) Kurama ridge, (C) *Malus sieversii* , (D) *Betula tianschanika*, (E) *Juglans regia* , (F) *Restella albertii*. Eastern Alay region: (G, H) Alay ridge, (I) *Caragana pleiophylla*, (J) *Dasiphora parvifolia*, (K) *Lonicera paradoxa*, (L) *Sorbus tianschanica* . South‐Chatkal: (M) Nanay of Hill, (N) *Prunus spinosissima*, (O) *Pistacia vera* , (P) *Hippophae rhamnoides* , (Q) *Crataegus tianschanica*, (R) *Zabelia corymbosa*.

The elevation of this region ranges from 357 to 3385 m above sea level, and its total area covers 2636 km^2^. Compared to other botanical–geographical regions within the districts, the Chorkesar region is the richest in tree and shrub species. Field studies conducted in this area recorded 103 species of trees and shrubs, accounting for 62.42% of the total species richness of the dendroflora of the Fergana Valley. The northwestern part of this region is bordered by the Kurama mountain range. The flora of this region is characterized by a composition of xerophytic tree and shrub species adapted to arid climatic conditions, such as *Juniperus seravschanica* , *J. turkestanica* , *Ephedra equisetina* , *Rosa canina* , *R. kokanica* , *Lonicera nummulariifolia*, and *Celtis caucasica* . In areas with sufficient moisture, typical representatives of broad‐leaved forests are also present, including *Juglans regia* , *Betula tianschanica*, *Malus sieversii* , *Acer semenovii*, *Cerasus mahaleb* , and other species (Figure 2). In this area, species listed in the National Red Book, such as *Restella albertii* and *Acantholimon margaritae*, are found (Figure 2). In the Chorkesar district, 15 species have been included in the IUCN Red List. Among them, *Betula tianschanica* (EN), *Malus niedzwetzkyana* (EN), and *Malus sieversii* (VU) are at risk of extinction. Species such as *Fraxinus sogdiana*, *Juglans regia* , *Juniperus seravshanica*, and *Populus pruinosa* Schrenk are considered globally near threatened (NT). Additionally, eight taxa—*Amygdalus petunnikovii*, *Celtis caucasica* , *Euonymus koopmannii*, *Crataegus korolkowi*, *Crataegus pontica*, *Restella albertii*, *Sorbus tianschanica* , and *Populus nigra* —are classified as of least concern (LC) (Eastwood et al. 2009; www.iucnredlist.org). Only two species in the flora of this area (*Restella albertii* and *Acantholimon margaritae*) are listed in the National Red Book of Uzbekistan and are protected by law (Figure 2).

#### 3.5.2 Eastern Alay BGR (Figure 1, I‐3‐b)

This region covers a total area of 3327 km^2^, with an elevation range of 441–3611 m above sea level. A total of 87 species of trees and shrubs (52.72%) were recorded in this area. During field studies, the highest level of biodiversity was recorded on the northern slopes of the Shakhimardan River basin, in the village of Iordan. Shakhimardan is a small exclave of the Republic of Uzbekistan, entirely surrounded by the Batken Region of the Kyrgyz Republic. This area is considered one of the regions of the Fergana Valley with the highest biological diversity, rich in rare, endemic, and relict species. The characteristic species composition of this area includes *Juniperus semiglobosa*, *Ephedra intermedia*, *Rosa beggeriana* , *R. laxa* , *Caragana alaica*, *C. pleiophylla*, *Clematis songorica*, *C. alpina subsp. sibirica*, *Dasiphora parvifolia*, *Lonicera microphylla*, *L. simulatrix* , *L. webbiana* (= *L. karelinii*), *Rhamnus coriacea*, *Spiraea pilosa*, *Sorbus tianschanica* , *Pyrus korshinskyi*, and other species (Figure 2). In addition, the area is home to *Lonicera paradoxa*, a rare and subendemic species (Figure 2). In the Eastern Alay region, certain species—*Pyrus korshinskyi* (CR), *Lonicera paradoxa* (EN), *Amygdalus bucharica* (VU), and *Malus sieversii* (VU)—are at risk of extinction. Species such as *Fraxinus sogdiana*, *Juniperus seravshanica*, and *Pistacia vera* are considered globally near threatened (NT). Meanwhile, *Celtis caucasica* , *Crataegus korolkowi*, *Sorbus tianschanica* , and *Populus nigra* are classified as least concern (LC), and one species, *Sorbaria olgae*, is listed as data deficient (DD) in the IUCN Red List, with its rarity status assessed (Eastwood et al. 2009; www.iucnredlist.org). *Lonicera paradoxa* is also included in the Red Data Books of Uzbekistan, Kyrgyzstan, and Tajikistan. Currently, research is being conducted by Daminova N. and Nosirov S. to conserve this species both ex‐situ and in situ in the wild (Daminova et al. 2024).

#### 3.5.3 South‐Chatkal BGR (Figure 1, I‐2‐a)

This region harbors 58 species (35.15%) of tree and shrub taxa. The elevation of the area ranges from 404 to 1859 m above sea level, and its total area covers 1518.9 km^2^. The peak of biodiversity was recorded in the northern part of the Fergana Valley, in the southern branches of the Chatkal mountain range, specifically in the lower sections of the Poshshaota basin, at elevations ranging from 1100 to 1500 m above sea level. Characteristic species of the region include *Rhamnus cathartica* , *Elaeagnus angustifolia* , *Hippophae rhamnoides* , *Pistacia vera* , *Atraphaxis compacta*, *A. spinosa* , *A. virgata* , *Rosa persica*, *Lonicera altmannii*, *L. korolkowii* , and *Prunus spinosissima* (Figure 2). Field studies conducted in 2020–2021 revealed the species *Crataegus tianschanica* and *Zabelia corymbosa* were discovered in the border areas of Nanay village, located in Yangikurgan district, near the Kyrgyz Republic. These species were recorded as new additions to the flora of Uzbekistan (Figure 2). Species such as *Amygdalus bucharica* (VU), *Fraxinus sogdiana*, *Pistacia vera* , *Populus pruinosa* (NT), *Populus nigra* , and *Zabelia corymbosa* (*Abelia corymbosa*) (LC) from the local flora are included in the IUCN Red List (Eastwood et al. 2009; www.iucnredlist.org).

According to research conducted by our research group during 2020–2023 in the Fergana Valley, the northern foothills and the Bozbu–Ungurtepа massif were identified as Important Plant Areas (IPAs) due to their high botanical value and special ecological significance (Tojibaev et al. 2025; Toledo et al. 2012; Van Heezik et al. 2014). The majority of these badlands are located within the Chorkesar region, while a smaller portion lies within the territory of the Chatkal region. A small part of the Bozbu–Ungurtepа massif is located within this region, while the majority lies within the territory of the Fergana Valley that belongs to the Kyrgyz Republic. It is especially important to emphasize that within the Chorkesar, Eastern Alay, and Chatkal regions, there are natural landscapes with high wild plant diversity that require conservation—particularly in the Chorkesar and Eastern Alay areas. These regions include habitats with populations of endangered species, endemic and relict plants, and plant communities of high botanical value.

#### 3.5.4 Western–Alay BGR (Figure 1, I‐3‐a)

This area encompasses 54 species of trees and shrubs, accounting for 32.7% of the total. The total area of the region is 743.62 km^2^, with an elevation range from 415 to 3013 m above sea level. The highest species richness coefficient corresponds to the natural landscapes located near the villages of Damersad, Nazar, Khushyor, and Surati in the Sokh district, which is part of this region. Within the flora of the region, tree and shrub species such as *Crataegus korolkowii*, *C. turkestanica* , *Caragana alaica*, *Ephedra equisetina* , *Hippophae rhamnoides* , *Prunus bucharica*, *P. verrucosa* , *Rosa ecae* , *R. fedtschenkoana* , *Spiraea hypericifolia* , and *Rhamnus coriacea* occur in a scattered distribution. Sokh district is an enclave of Uzbekistan located within the territory of Batken Region of the Kyrgyz Republic, surrounded by the Alay mountain ranges. During research conducted in the Shorsuv hills of the Western Alay district, new populations of *Calligonum calcareum* were discovered. This species is one of the rare and endemic plants listed in the National Red Book and is classified as Critically Endangered (CR) according to the IUCN Red List and its criteria (Eastwood et al. 2009; www.iucnredlist.org).

#### 3.5.5 Kayrakum–Yazyavan BGR (Figure 1, II‐1‐a)

The total area of this region is 6137.6 km^2^, with an elevation range from 396 to 883 m above sea level. The flora of the region includes 53 species of the dendroflora, accounting for 32.1% of the total. The peak of species diversity was recorded in the central part of the Fergana Valley, within the Yazyavan desert at elevations ranging from 350 to 420 m above sea level. The peak of species diversity was recorded in the central part of the Fergana Valley, at elevations ranging from 350 to 420 m above sea level in the Yazyavan desert. In the flora of the region, xerophytic trees and shrubs adapted to arid desert climatic conditions are represented by species such as *Calligonum arborescens C. caput–medusae*, *C. litwinowii*, *Caragana halodendron*, *Haloxylon ammodendron*, *H. Persicum*, *Halostachys caspica*, *Nitraria schoberi*, *Lycium dasystemum*, *L. ruthenicum*, *Xylosalsola arbuscula*, *X. paletzkiana*, *X. richteri*, *Populus pruinosa* among the chamaephytes, species like *Astragalus cognatus*, *Halocnemum strobilaceum* are found. Rare and endemic species such as *Calligonum calcareum* and *C. leucocladum* also occur. *C. calcareum* and *C. elegans* are included in the National Red Book. Their conservation status has also been assessed according to the IUCN Red List and its criteria (Eastwood et al. 2009; www.iucnredlist.org). In studies conducted on the flora of the region, no populations of *C. calcareum* were found at the site where the type specimen was originally collected. The main reason for this is that the area has been subjected to intense anthropogenic pressure. In particular, the natural habitats of the species have been degraded due to the construction of new residential buildings for the local population.

The lowest levels of taxonomic richness were recorded in Eastern Fergana (19 species, 11.51%), Arashan (28 species, 16.9%), and Kurama (15 species, 9%) botanical‐geographical regions.

The total area of the Eastern Fergana (Figure 1, II‐1‐b) region is 3207.3 km^2^. This area is entirely occupied by human settlements and is characterized by the absence of natural landscapes. During the field studies, dendroflora species were mainly recorded around the banks of the Naryn and Karadarya rivers. The total area of the Arashan (Figure 1, I‐1‐c) region is 927.66 km^2^, with an elevation range from 1200 m at the lowest point to 3900 m at the highest. The Kurama (Figure 1, I‐1‐d) region is geographically and administratively part of the Tashkent region. Its total area is 3333.1 km^2^. However, an area of approximately 5 ha in the part of this region bordering Namangan Region is administratively part of the Pop District of Namangan Region in the Fergana Valley. The elevation range of this area is from 1400 to 1900 m. Field studies were conducted within a 5‐ha area, and the results were analyzed based on the data obtained. The next study dedicated to the dendroflora of Uzbekistan will provide detailed information on the tree and shrub taxa of the Arashan and Kurama regions.

### 3.6 Distribution by Altitude Regions

In the Uzbekistan part of the Fergana Valley, representatives of the dendroflora are distributed across an elevation range of nearly 300–3200 m, encompassing desert, foothill, mountain, and alpine meadow zones. Among them, the tugai forests located in the central part of the valley hold a special place. The majority of the species included in the dendroflora have a wide distribution range along the elevation gradient. According to their bioecological and ecogeographical characteristics, they grow in various elevation zones and exhibit specific patterns of distribution along these altitudinal gradients.

**FIGURE 3:**
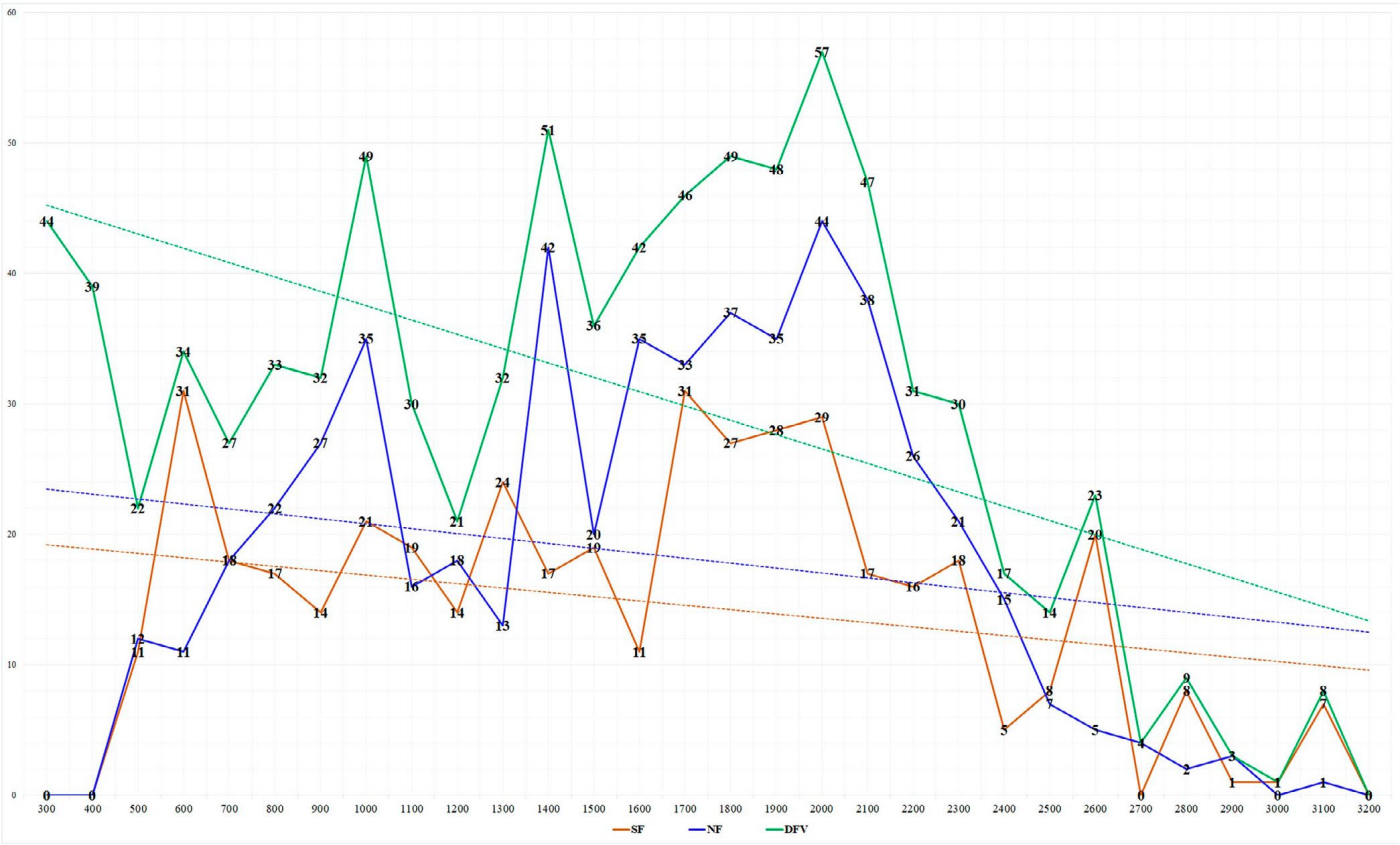
Altitudinal distribution of dendroflora species at 100‐m intervals.

The distribution of dendroflora species across elevation zones was carried out based on the classification proposed by Academician Zakirov (1947) for Central Asia. As a result of analyzing the altitudinal distribution of the dendroflora of the Fergana Valley, the following findings were obtained. Among the elevation zones, the mountain zone (1500–2700 m) is dominant, with 112 species represented in the dendroflora. In descending order of species richness, the subsequent zones are the foothill zone (500–1500 m, 101 spp.), the desert zone (300–500 m, 53 spp.), and the alpine meadow zone (2800–3200 m, 17 spp.). In addition, to identify areas of high biodiversity within each geomorphological level, changes in species composition were studied at 100 m elevation intervals. According to the results, the 2000–2100 m range within the mountain zone was found to be the most species‐rich, with a total of 57 species. The next highest levels of species richness were observed, with a relatively small difference, in the 1400–1500 m range of the foothill zone (51 spp.) and the 300–400 m range of the desert zone (44 spp.), indicating high dendroflora diversity in these areas. The highest value recorded in the alpine meadow zone corresponds to the 2800–2900 m range, where 9 species were identified (Figure 3).

To compare species composition and enhance the accuracy of the results, two contrasting macroexposures within the study area—Northern Fergana (NF) and Southern Fergana (SF)—were selected. The analysis focused on the geomorphological zones accepted as units of comparison, with the aim of identifying similarities and differences in species composition based on the following:

1. Species richness along the elevation gradient and its changes at 100‐m intervals.
2. Taxonomic diversity within each geomorphological zone.
3. Vertical and horizontal distribution of tree and shrub taxa (Rosaceae, Cannabaceae, Elaeagnaceae, Rhamnaceae, and Ulmaceae).

The results along the elevation gradient showed that at elevations of 600–700 m (Shorsuv, Chimgan, Asaka Hill, and Shirmonbulak (due to the genera *Lonicera*, *Prunus*, *Tamarix* (4 species each), *Rosa* (3 spp.), *Zygophyllum* and *Atraphaxis* (2 species each)), and 2600–2900 m (Shakhimardan (*Lonicera nummulariifolia*, *L. paradoxa* , *L. simulatrix* , *L. webbian*, *Prunus alaica*, *P. prostrata* var. *concolor*, *P. verrucosa* , *Cotoneaster multiflorus* , *C. pseudomultiflorus*, *Berberis oblonga*, *B. integerrima*, *Spiraea lasiocarpa*, *S. pilosa* , *Juniperus semiglobosa*, *Betula tianschanica*, *Salix iliensis and other species*)), the dendroflora of SF exhibited higher species richness compared to NF (Figure 3). In the remaining elevation transects—particularly in the upper foothills of Southern Chatkal at 900–1100 m (*Prunus* (7 spp.), *Atraphaxis*, *Rosa* (4 species each), *Ephedra*, *Crataegus*, *Populus* (2 species each) *and other genera*) and 1400–1500 m ( *Prunus cerasifera* , *P. prostrata* , *P. erythrocarpa*, *P. verrucosa* , *P. spinosissima* , *P. verrucosa* , *Rosa kokanica* , *R. canina* , *R. fedtschenkoana* , *R. beggeriana* , *R. ecae* , *Lonicera humilis*, *L. korolkowii* , *L. microphylla* , *L. nummulariifolia*, *Crataegus turkestanica*, *C. songarica* , *C. tianschanica* , *and other species*), as well as in the 1600–2500 m (*Prunus* (9 spp.), *Salix*, *Rosa* (8 species each), *Lonicera* (5 spp.), *Crataegus*, *Astragalus*, *Acantholimon* (8 species each) *and other genera*) range of the Kurama mountain—the NF macroexposure showed greater species richness (Figure 3).

**FIGURE 4:**
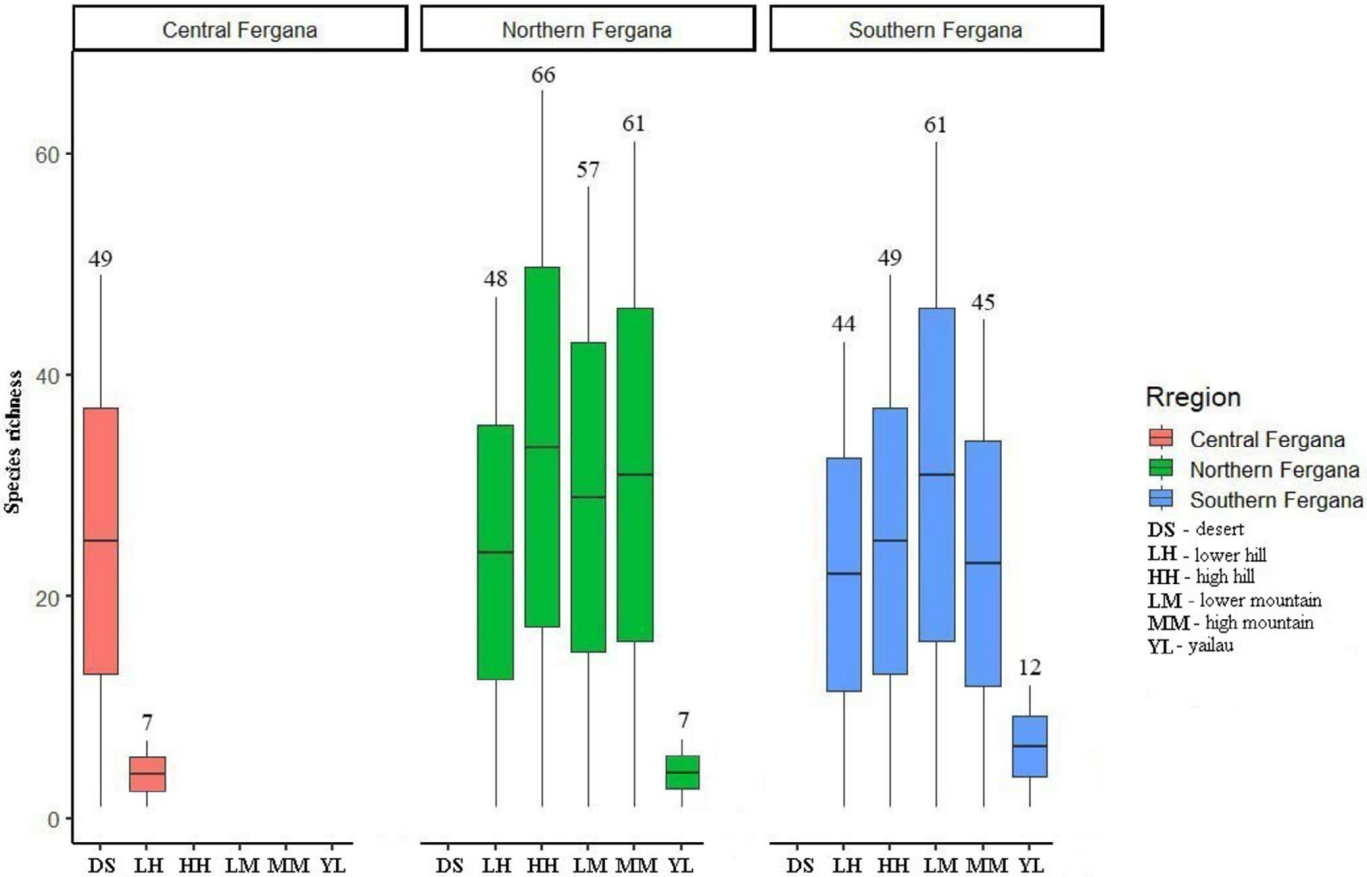
Fergana Valley: Species diversity indices in the northern, southern, and central regions.

In terms of taxonomic diversity within the designated geomorphological zones, the NF macroexposure showed clear dominance. This is particularly evident in the number of species in the lower foothill (48 ≥ 44), upper foothill (66 ≥ 49), and mid‐mountain (61 ≥ 41) zones, where NF consistently surpasses SF. With a relatively small difference, SF showed dominance in the lower mountain (57 ≤ 61) and alpine meadow (7 ≤ 12) zones (Figure 4). In the dendroflora of NF, 119 species of trees and shrubs are found, accounting for 72.12% of the total. Of these, the lower foothill zone accounts for 29.09% of the total dendroflora (48 species), the upper foothill zone for 40% (66 species), and the mid‐mountain zone for 36.96% (61 species). In the lower foothill zone, the genera *Prunus* (7 spp./ 4.24%), *Atraphaxis* (5 spp./3.03%), *Rosa* (4 spp./2.42%) dominate; in the upper foothill zone, *Prunus* (7 spp./4.24%), *Rosa* (7 spp./4.24%), *Atraphaxis* (5 spp./3.03%), *Crataegus*, *Lonicera* (each with 4 species/2.42%) prevail; and in the mid‐mountain zone, *Prunus*, and *Rosa* (each with 7 species/4.24%), *Acantholimon*, *Lonicera*, and *Salix* (each with 6 species/3.63), *Astragalus* (4 spp./2.42%), and *Crataegus* and *Cotoneaster* (each with 3 species/1.81%) lead the species composition in NF.

The dendroflora of SF comprises a total of 106 species, accounting for 64.24% of the entire dendroflora. The geomorphological zones where SF predominates are the lower mountain zone (61 spp./36.96%) and the alpine meadow zone (12 spp./7.24%). The dominance of the lower mountain zone is mainly due to the genera *Rosa* (9 spp./5.45), *Prunus* and *Lonicera* (each with 6 species/3.63%), *Astragalus* (sect. *Macrothrix* and *Xiphidium*), *Crataegus*, *Cotoneaster*, *Clematis*, and *Spiraea* (each with 3 species/1.81%). In addition, the dominance of SF in the alpine meadow zone is attributed to the presence of *Lonicera* (3 spp./1.81%), *Acantholimon* (2 spp./1.21%), and other genera.

**FIGURE 5:**
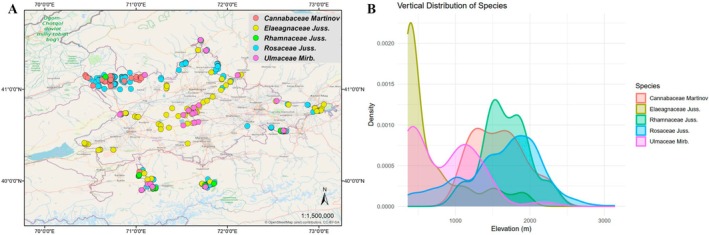
(A) Horizontal and (B) vertical distribution of Rosaceae, Cannabaceae, Elaeagnaceae, Rhamnaceae, and Ulmaceae.

The study identified the distribution along vertical and horizontal gradients of five families, covering the richest (Rosaceae) and least diverse families (Cannabaceae, Elaeagnaceae, Rhamnaceae and Ulmaceae) of the area. Regarding the vertical and horizontal structure of the area, 48 species belonging to 16 genera from five families were recorded, showing an uneven distribution of all taxa across vertical and horizontal forest levels. The Rosaceae family is represented by the highest number of genera and species in the dendroflora of the Fergana Valley. Species of this family were recorded across all zones of the study area—desert, foothill, mountain, and alpine meadow—within an elevation range of 300 to 3100 m above sea level. In terms of species richness across elevation ranges, the highest concentration was observed between 1400 and 2100 m. In contrast, tree and shrub species were found to be sparsely distributed in the 2700 to 3200 m range (Figure 5). The families Cannabaceae (1000–2300 m a.s.l.), Elaeagnaceae (300–2000 m a.s.l.), Rhamnaceae (1000–2200 m a.s.l.), and Ulmaceae (300–2200 m a.s.l.) occur within particular elevational ranges.

The species composition of the Cannabaceae and Ulmaceae families remained similar along horizontal gradients, but the species composition of the Rosaceae, Elaeagnaceae, and Rhamnaceae families changed along vertical gradients. Some species are restricted to vertical zones, perhaps because they have not been able to expand beyond their natural habitats or because they have been subjected to strong anthropogenic pressure. For example, Pyrus korshinskyi was reported from southern Uzbekistan (based on herbarium specimens collected by A. Regel in 1883; Litwinow, 1902 (https://powo.science.kew.org (May 15, 2023)), where it is thought to have grown on the slopes of the Western Tashbulak Mountains to an altitude of 4000 ft. At present, this species is found only in the lower montane region (ca. 1000–1800 m). Some tree and shrub taxa are quite limited in their vertical distribution (e.g., *Cotoneaster suavis* 2200–2300 m a.s.l., *Crataegus chlorocarpa* 1700–1800 m a.s.l., *Rosa laxa* 2000–2100 m a.s.l., and *Crataegus tianschanica* 1400–1500 m a.s.l.). In addition, a number of plant taxa show significant vertical ranges (Rosa persica 800–1100 m a.s.l., *Hedlundia persica* 1300–1500 m a.s.l., and *Rhamnus cathartica* 1400–1900 m a.s.l. at medium altitudes; Cotoneaster pseudomultiflorus 1800–2400 m a.s.l., *Sorbus tianschanica* 2000–2400 m a.s.l., and *Dasiphora parvifolia* 1800–2600 m a.s.l. at medium and high altitudes). The differences in vertical amplitudes are probably related to the increase in temperature in the regions of the Turanian Lowlands. *Crataegus turkestanica* occurs throughout the vertical transect.

**FIGURE A1:**
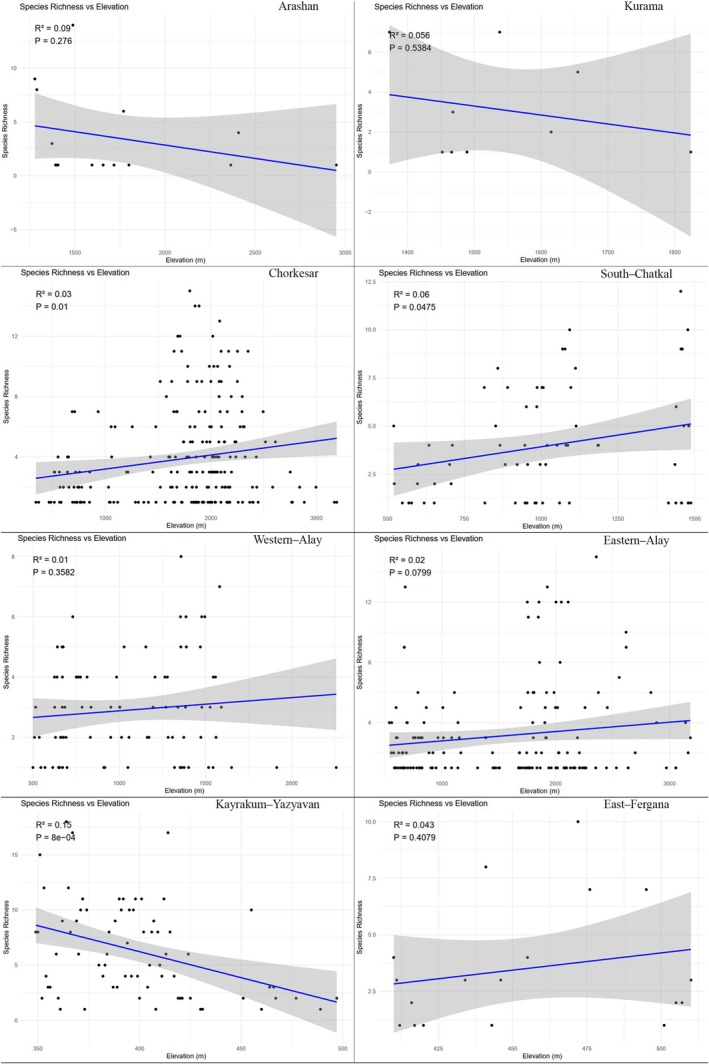
The relationship between species richness and elevation in botanical–geographical regions.

To determine the relationship between species richness and elevation across botanical‐geographical regions, as well as to assess whether species composition differs significantly between them, statistical analysis was performed using the R software. According to the analysis results, the coefficient of determination for the Arashan region was *R* ^2^ = 0.09 (Figure A1). This indicates that 9% of the variation in species richness is related to elevation, while the remaining 91% is likely influenced by other factors such as moisture, temperature, soil characteristics, or anthropogenic impact. The *p*‐value obtained from the PERMANOVA analysis was *p* < 0.276, indicating that the relationship is not statistically significant. Also, indicates that elevation does not have a statistically significant effect on species composition. The results showed the following values: Kurama—*R* ^2^ = 0.056, *p* < 0.5384; Western Alay—*R* ^2^ = 0.01, *p* < 0.3582; Kayrakum–Yazyavan—*R* ^2^ = 0.15, *p* < 8e‐04; and Eastern Fergana—*R* ^2^ = 0.043, *p* < 0.4079 (Figure A1). These findings indicate a very weak relationship between elevation and species composition, and in most cases, the relationship is statistically unreliable. This suggests that elevation is not the primary factor influencing species composition. Chorkesar showed a coefficient of determination of *R* ^2^ = 0.03 with a *p*‐value of 0.01, while Eastern Alay recorded *R* ^2^ = 0.02 and *p* < 0.05 (Figure A1). In this case, the *R* ^2^ coefficients indicate a noticeable but weak influence of elevation on species composition, accounting for only 3% or 2% of the variation. However, the results are statistically significant, indicating that there is a somewhat reliable relationship between elevation and species richness. Nevertheless, the ecological impact of this factor is very weak. From this, it can be concluded that although the *R* ^2^ coefficient is low, it indicates a statistically significant effect. In other words, elevation does not play a primary role in determining species richness, but it does have a noticeable influence.

Southern Chatkal showed a result of *R* ^2^ = 0.06 and *p* < 0.0475 (Figure A1). This indicates that elevation has a weak, or ecologically weak to moderate, influence on species composition—it is not entirely insignificant. The *p*‐value indicates statistical significance, as it is approximately equal to the PERMANOVA threshold of *p* < 0.05. This suggests that the observed relationship is not random and can be considered statistically reliable. Thus, this factor (elevation) has a real and significant influence on species composition. Based on the results above, it can be concluded that although the effect of elevation on species richness in the Chorkesar, Eastern Alay, and Southern Chatkal regions is ecologically weak to moderate, it is statistically significant.

### 3.7 Rare and Threatened Taxa

In the Fergana Valley (Uzbekistan part), 25 tree and shrub species have been included in the IUCN Red List. These species are under threat due to intense anthropogenic pressure and have been added to the IUCN database based on various criteria. Among them, eight are considered threatened species (2 CR—Pyrus korshinskyi, Calligonum calcareum; 4 EN—Betula tianschanica, Calligonum elegans, Lonicera paradoxa, Malus niedzwetzkyana; 2 VU—Amygdalus bucharica, *Malus sieversii* ). Additionally, five species have been classified as globally near threatened (NT). Furthermore, 11 tree and shrub taxa have been categorized as least concern (LC), and one species is listed as data deficient (DD), indicating a lack of sufficient information for assessment. Accordingly, the share of threatened and potentially threatened tree and shrub taxa in the Fergana Valley constitutes approximately 15.15% of the total dendroflora taxa. However, seven tree taxa (or 4.2% of all taxa) have not yet been assessed.

This study revealed that there are no dendroflora species endemic to the Uzbek part of the Fergana Valley. However, the flora of the study area includes a few noteworthy and rare subendemic taxa (*Acantholimon margaritae*, *Calligonum calcareum*, *Calligonum elegans*, *Lonicera paradoxa*, *Restella albertii*). These species are considered rare and endemic according to various criteria of the National Red Book of Uzbekistan (Khasanov 2019). The proportion of rare, endemic, and subendemic taxa makes up 3.1% of the total dendroflora.

### 3.8 Threats

For the dendroflora of the Fergana Valley, the majority of recorded species are subject to at least three of the following threats. These threats are grouped into 10 main categories:

1. Population growth and increasing demand for housing.
2. Rising levels of urbanization and development of tourism.
3. Accelerated rates of economic growth.
4. Conversion of natural landscapes for agricultural cultivation and residential development.
5. Exploitation (logging) and destruction of natural habitats.
6. Continuous grazing by livestock.
7. Extraction of natural resources.
8. Construction of new roads and railways.
9. Expansion of fish farming and artificial lakes.
10. Sand extraction in the Kayrakum and Yazyavan deserts for construction materials, which is causing a sharp decline in both the species composition and distribution range of trees and shrubs in the area.

In particular, the tree and shrub composition of the Kayrakum–Yazyavan region (45 taxa = 27.7%) is exposed to multiple threats, including the following:

– Agricultural land conversion.
– Construction of new residential areas.
– Establishment of new economic zones.
– Sand extraction for construction materials.
– Continuous grazing by livestock.
– Construction of new roads.
– An increasing number of artificial lakes for fish farming.
– Habitat destruction due to tree and shrub cutting.

**FIGURE A2:**
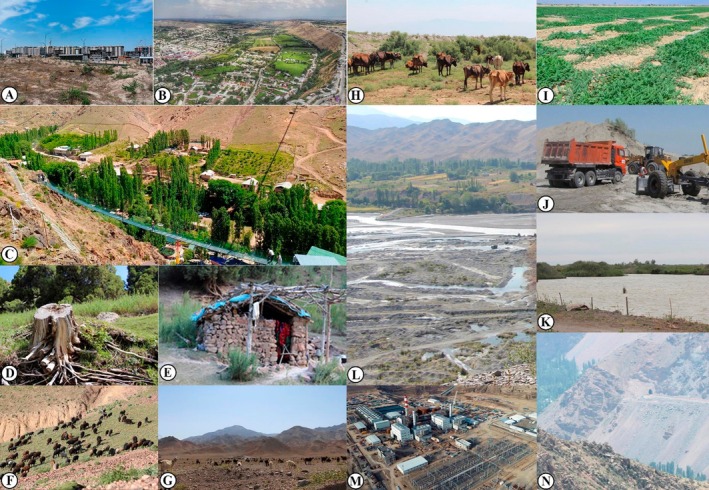
Main threats contributing to the decline of trees and shrubs: (A, B) Transformation of natural landscapes in the Eastern Alay region, (C–F) development of tourism, cutting of trees and shrubs, and continuous livestock grazing in the Chorkesar region, (G, L) unregulated livestock grazing in the Western Alay region, Extraction of gravel and sand from the Sokh River, (H–K) continuous livestock grazing in the Kayrakum–Yazyavan region, cultivation of agricultural crops on the Yazyavan sand massif, sand extraction from the Ulugnor sand massif, and an increasing number of artificial lakes for fish farming on the Yazyavan sand massif, (M) the newly constructed thermal power plant in the South–Chatkal region, (N) construction of new railway lines.

These factors have significantly impacted the survival and distribution of woody plant species in the region (Figure A2). In addition, 19 taxa (11.5%) growing in the Eastern Fergana region are subject to threats such as population growth and increasing demand for housing, rising levels of urbanization, and the complete transformation of natural landscapes. As a result, their habitats have been severely degraded. In the Chorkesar (103 taxa = 62.42%) and Eastern Alay (87 taxa = 57.72%) regions, which have high biodiversity coefficients, the tree and shrub species are experiencing habitat degradation due to several factors. For example, *Sorbaria olgae*, which occurs exclusively in the flora of the Eastern Alay region, has experienced habitat degradation as a result of the exploitation of trees and shrubs for fuelwood by the local population and the continuous grazing of livestock. *Lonicera paradoxa* is also one of the plant species endemic to this region. The natural habitats of this species have been drastically reduced due to the factors mentioned above. Such examples can be provided for several tree and shrub species. These include the leasing of forest lands to local populations, continuous livestock grazing, cutting for firewood, the development of tourism, and the construction of new recreational facilities. Such conditions are observed across all geographical regions of the Fergana Valley.

## 4 Discussion

A large part of the study area falls within the desert zone and has a continental (arid) climate. As a result, it is not rich in tree and shrub species. Despite the arid climate, the area encompasses 165 species of tree and shrub taxa. According to Eastwood et al. (2009), there are approximately 500–600 species of tree and shrub taxa in Central Asia, and about 33% or 27.5% of them are found in the study area. This fact confirms that the dendroflora of the Fergana Valley is characterized by remarkable diversity. Based on the study of scientific sources written as a result of more than a century of research conducted in different parts of the Fergana Valley, as well as field observations carried out within this research framework, the first complete list of native dendroflora growing in the natural landscapes of the Fergana Valley (Uzbekistan) was compiled. This list includes 165 species of trees and shrubs belonging to 60 genera and 32 families. Of these, 2 species are invasive ( *Ulmus glabra* and *Rubus idaeus* ), and 5 species are listed in the national Red Book (*Acantholimon margaritae*, *Calligonum calcareum*, *C. elegans* , *Lonicera paradoxa* and *Restella albertii*). Rare and subendemic tree taxa make up 3.03% of the total dendroflora.

In terms of taxonomic diversity, the families with the highest number of taxa are Rosaceae (42 species), Fabaceae (15 species), Amaranthaceae and Salicaceae (14 species each), and Polygonaceae (11 species). The most species‐rich genera include *Rosa* (11 spp.), *Acantholimon*, *Prunus*, and *Salix* (each with 10 spp.). Studies focusing on tree and shrub flora show that the Rosaceae family dominates the spectrum of polymorphic families, followed by the Fabaceae family. For instance, the studies by Yurukov and Zhelev (2001), Abbate et al. (2012), Roma‐Marzio et al. (2016), Médail et al. (2019), and Meddour et al. (2021) have highlighted Rosaceae as the richest family of tree and shrub species in the Mediterranean‐European countries. Similarly, research by Palgrave et al. (2007), Gillet and Doucet (2012), Beech et al. (2017), and Meddour et al. (2021) noted that Fabaceae is one of the most diverse families globally and ranks second after Rosaceae in terms of richness in tree and shrub species. The results of this study are consistent with these findings.

The floristic elements are very diverse, comprising seven main elements. The majority of trees, 97 taxa (58.79%), belong to the Central Asian and 48 taxa (29.1%) to the Temperate Asian floristic element. On the one hand, the flora of this region is predominantly characterized by temperate elements, a pattern consistently supported across the family, genus, and species levels. This temperate character (Types 8–14) is further substantiated through a comparative analysis with previously published findings on woody plant flora from both northern and southern regions of China (Li et al. 2017; Liu et al. 2024). The results highlight the close relationship between the dendroflora of Temperate Asia and Central Asia, as well as the importance of regional endemism, emphasizing the need to conserve unique and endemic species as key components of biodiversity. Moreover, they demonstrate the distinctiveness of the region's biogeographic structure.

Among the life forms of tree and shrub taxa, the most represented group is the nanophanerophytes of the phanerophyte category (84 species, making up 50.90%). This is explained by the richness of the forests in the study area in single‐ or multi‐stemmed shrub taxa such as *Rosa* (11 species), *Lonicera* and *Tamarix* (8 species each), *Prunus* (7 species), *Calligonum* (6 taxa), *Cotoneaster* and *Atraphaxis* (5 species each). The proportion of microphanerophyte and mesophanerophyte species is low. Chamaephytes (30 species) are represented by a small number of species. Tree and shrub taxa are found in several parts of the natural landscapes of the Fergana Valley, but most of them are located in the evergreen and broad‐leaved forests of Chorkesar (northern part of the valley) and Eastern Alay (southern part). From both regions, species richness gradually decreases toward the central part of the valley. This is associated with the desert zone located in the central part of the valley and its arid climatic conditions, where desert xerophytes have formed a unique habitat. In terms of botanical–geographical distribution, the regions with the highest proportion of species richness are Chorkesar (62.42%), Eastern Alay (52.72%), and Southern Chatkal (35.15%), while the lowest indicator was found in Eastern Fergana (11.51%). The populations of rare and subendemic species cited for the study area were found in Chorkesar (*Acantholimon margaritae*, *Restella albertii*), Eastern Alay (*Lonicera paradoxa*), Western Alay (*Calligonum calcareum*), and Kayrakum–Yazyavan (*Calligonum elegans*). In addition, 15 species included in the IUCN Red List were identified in Chorkesar, 10 in Eastern Alay, 7 in Southern Chatkal, and 5 in Western Alay. Also, species listed in the IUCN Red List were found in Arashan, Kurama, Kayrakum–Yazyavan (4 species each), and Eastern Fergana (3 species), although they represent a lower proportion.

The dendroflora of the Fergana Valley was analyzed for species richness along an altitudinal gradient at 100 m intervals. The recorded taxa occur between 300 and 3200 m a.s.l., revealing a heterogeneous distribution of species richness across elevation zones. The highest diversity of tree and shrub taxa was observed in the montane ecosystems, particularly between 2000 and 2100 m, where climatic conditions are most favorable for dendroflora development (Kharkwal et al. 2005; Manish et al. 2017). In contrast, species richness declined markedly in steppe, desert, and high‐mountain grassland zones. At subalpine elevations, only taxa adapted to severe climatic conditions—mainly low evergreen trees and shrubs—were recorded (Kharkwal et al. 2005; Manish et al. 2017; Mehrabian et al. 2020). A comparative analysis of the northern (NF) and southern (SF) exposures showed that NF dendroflora predominated in the lower, mid‐, and upper montane belts, while SF dendroflora dominated in the lower montane and foothill–pasture zones. This pattern can be explained by the broader geomorphological extent of NF slopes (3052 km2) and the wide distribution range of genera such as *Prunus*, *Rosa*, *Atraphaxis*, *Crataegus*, and *Lonicera*. Species diversity within the families Rosaceae, Cannabaceae, Elaeagnaceae, Rhamnaceae, and Ulmaceae was analyzed along both horizontal and vertical gradients. The Rosaceae family exhibited the highest diversity (42 taxa), particularly within the genus Rosa (11 taxa), concentrated at elevations of 1400–2100 m. Several genera within this family also displayed wide vertical distribution ranges. Compared to Cannabaceae, Elaeagnaceae, Rhamnaceae, and Ulmaceae, the Rosaceae family demonstrated significantly greater variation in species richness across both elevation and exposure gradients. As highlighted by Di Biase et al. (2021), altitudinal variation—accompanied by shifts in temperature, precipitation, and soil properties—exerts a strong positive influence on biological diversity. Mountain ecosystems thus provide valuable contexts for examining how environmental factors shape ecological dynamics. The findings of this study are consistent with the conclusions of Di Biase et al. (2021). However, at elevations above 2600 m, increased precipitation, higher soil moisture, and persistent winds were found to limit the distribution of several species within the study area.

Statistical analyses revealed that elevation has a significant, albeit weak, effect on species richness. In particular, in the Chorkesar and Eastern Alay regions, the observed correlation values accounted for only 2%–3% of the total variability. Nevertheless, these results were statistically significant, confirming that there is a modest but consistent relationship between elevation gradients and species richness. In contrast, in the Arashan, Kurama, Western Alay, Kayrakum–Yazyavan, and East–Fergana biogeographic regions, the relationship between elevation and species composition was very weak and statistically unreliable, suggesting that elevation is not a primary driving factor. This implies that species distribution in these areas is more likely influenced by other factors, such as moisture, temperature, soil properties, or anthropogenic impacts. Overall, these findings underscore the heterogeneous nature of plant community organization across the region and highlight the importance of incorporating local ecological and biogeographic contexts when developing conservation strategies.

In the study area, 25 taxa have been included in the IUCN Red List, of which five are also listed in the National Red Book of Uzbekistan. Eight species have been evaluated as endangered or threatened according to IUCN criteria. In the central part of the Fergana region, large areas of the Kayrakum, Akkum (Yazyavan), and Karakalpak deserts have been extensively transformed in recent years due to agricultural expansion, construction, and fish farming activities, resulting in significant degradation of natural landscapes. Consequently, the natural habitats of species belonging to the families Polygonaceae (e.g., *Calligonum*) and Amaranthaceae (*Haloxylon*, *Xylosalsola*), among others, have been severely reduced, placing endemic species such as *Calligonum* elegans and *C. calcareum* at high risk of extinction. In the Chorkesar region, *Restella albertii*, included in the National Red Book of Uzbekistan, has been recorded, along with *Malus sieversii* , *Prunus petunnikowii*, *Lepidolopha komarowii*, and *Acantholimon korolkovii*, which are listed in the Red Books of Kyrgyzstan and Tajikistan. Populations of *Betula tianschanica* occur at elevations of 2000–2200 m in the Chodaksai and Kainlisai basins of the Kurama mountain; however, their natural area has decreased by 0.5–1.0 ha over the past two decades. The village of Shakhimardan, located in the Eastern Alay region, is characterized by high species diversity and a notable richness of endemic and relict species. A total of 21 rare and threatened plant species have been recorded in this area. The subendemic species *Lonicera paradoxa* serves as an important floristic link between the Shakhimardan River basin and the Turkestan and Zarafshan mountain ranges. The area also harbors regionally threatened species such as *Pyrus korshinskyi*, *Malus sieversii* , and *Clematis alpina subsp. sibirica*, the latter of which occurs in Uzbekistan only in the Alay part of the Pamir–Alay mountain system. Around Shakhimardan, birch stands (*Betula tianschanica* ( *B. turkestanica* )) under strong anthropogenic pressure are confined to elevations of 1900–2000 m along both banks of the Dugobasai valley, where only 14 mature birch trees remain. These findings highlight the importance of conserving the dendrofloral diversity of the Fergana Valley, particularly its endemic, relict, and threatened species.

## 5 Conclusions

The checklist reflecting the taxonomic structure of the Fergana Valley dendroflora is of great scientific importance, as it serves as a foundation for the inventory and conservation of plant biodiversity in specific regions, and it accurately represents the diversity of tree and shrub species characteristic of the valley. The main biodiversity hotspots of tree and shrub species are located in the Chorkesar and Eastern Alay biogeographic regions (BGR), which fully meet the A and B category criteria of Important Plant Areas (IPA). In addition, there are several areas of high botanical value, including the Angren Plateau, the upper reaches of the Chodak and Chorkesar rivers, the Pap foothills, Karatag, Ungor Tepa, the Chartak foothills, the Akkum sands, the upper Syrdarya basin, the Teshiktosh foothills, the Chilustun and Kirtashtau Mountains, Shakhimardan, and Sokh. Therefore, the inventory of tree and shrub flora, biodiversity conservation, and protection of wild populations in these regions hold both scientific and practical significance. The comprehensive data obtained in this study—particularly regarding the taxonomic composition and distribution of tree and shrub species within the natural vegetation cover of the Fergana Valley, as well as rare and endemic species with restricted ranges—make a significant contribution to filling existing knowledge gaps. Moreover, the results of this study may serve as an important scientific resource for future monitoring projects aimed at “grid‐based mapping of the flora of the Fergana Valley,” as well as for the planning of regional conservation measures. From a biogeographical perspective, these findings can help identify the rarest and most ecologically significant tree species requiring protection. The conservation of certain dendroflora taxa—such as *Betula tianschanica*, *Malus sieversii* , *Pyrus korshinskyi*, *Juglans regia* , *Pistacia vera* , *Amygdalus bucharica*, *Sorbus tianschanica* , *Populus pruinosa*, *Calligonum calcareum*, *C. elegans* , *Lonicera paradoxa*, and *Restella alberti*—in their natural habitats necessitates the prompt and effective implementation of appropriate conservation measures. While this process may be complex, it is undoubtedly worthwhile in the pursuit of biodiversity preservation.

## Author Contributions

**Nazokat Daminova:** conceptualization (lead), data curation (lead), formal analysis (lead), methodology (lead), resources (equal), software (lead), supervision (lead), validation (equal), visualization (lead), writing – original draft (lead), writing – review and editing (lead). **Xian‐Han Huang:** conceptualization (supporting), formal analysis (supporting), methodology (supporting). **Hushbaht R. Hoshimov:** methodology (supporting), software (supporting). **Dilmurod Makhmudjanov:** methodology (supporting), writing – review and editing (supporting). **Farkhod I. Karimov:** investigation (supporting), writing – original draft (supporting). **Hee‐Young Gil:** funding acquisition (equal), writing – review and editing (supporting). **Komiljon Sh. Tojibaev:** funding acquisition (equal), investigation (supporting), project administration (lead), writing – review and editing (supporting). **Hyeok Jae Choi:** funding acquisition (lead), investigation (supporting).

## Funding

This study, conducted as part of the CABCN and Central Asia Green Road Project III, was supported by the Korea National Arboretum (Project No. KNA1‐249‐25‐2) and the Korea Basic Science Institute (National Research Facilities and Equipment Center) under grant (Grant No. 2023R1A6C101B022) funded by the Ministry of Education. Additionally, the study was supported by the State Program “Digital Nature: Development of a Digital Platform for the Flora of Central Uzbekistan” (2025–2029), implemented by the Institute of Botany, Academy of Sciences of the Republic of Uzbekistan, as well as by the projects “Taxonomic Revision of Polymorphic Families in the Flora of Uzbekistan” (A‐FA‐2021‐427) and “Preparation of the First Uzbek‐Language Edition of the Flora of Uzbekistan” (AL‐9224104450).

## Conflicts of Interest

The authors declare no conflicts of interest.

## Data Availability

The original contributions presented in this study are included in the article. Further inquiries can be directed to the corresponding author.

## Appendix A

**TABLE A1:** Field visits during the present study (2020–2023).

T/n	Exact location	Covernorate	Latitude (N)	Longitude (E)	Date
1	Nanay hill		41.537064	71.704610	May 17, 2020 July 24, 2021
2	Gowasay		41.156335	71.094239	May 18, 2020
3	Imam‐Ata mountain		40.548117 40.548745	72.610435 72.605079	May 21, 2020 May 15–16, 2021
4	Syrdarya river		40.72448 40.722379 40.73962	70.821587 70.828099 70.883028	June 9–10, 2020 May 25, 2021 August 3, 2021
5	River basin Chadaksay (Kandagansay;		41.110830 41.134693 41.120256 41.095164	70.708324 70.735079 70.738662 70.714891	June 15–16, 2020 May 6–7, 2021 July 6–9, 2021 June 26, 2022
	Kainlisay;		41.101894	70.632179	July 4, 2020
	Sansalaksay;		41.105673 41.108388 41.100973	70.602175 70.681220 70.593273	July 5–6, 2020 May 7, 2021 June 23, 2022
	Indagansay;		41.147525	70.700638	July 8, 2021
	Tavaksay and Suyuksu;		41.143623	70.746658	July 9, 2021
	Phashshaxanasay;		41.098057 41.104790 41.106115	70.627464 70.599720 70.614148	May 8, 2021 July 10, 2021 June 22, 2022
	Uriklisay;		41.061691	70.772549	July 11, 2021
	Betegal;		41.138343	70.597833	June 24, 2022
	Duob village)		41.073437	70.715349	June 25, 2022
6	Parda‐Tursun		41.144473	70.715988	June 17, 2020
	(Kenkulsay)		41.097905 41.09413	70.864542 70.853256	May 19, 2021 July 12, 2021
8	River basin Sox (Hushyar village;		39.898068	71.058394	September 19, 2020
	Surati mountain		40.022131 40.062307	71.166551 71.03214	September 20, 2020 May 18, 2022
	Damersat village		39.933036	71.177768	May 31, 2021
	Malmuth mountain		40.016880	71.188336	May 21, 2022
	Nazar village		39.913277	71.183501	May 20, 2022
	Tul mountain		40.117075	71.071425	May 19, 2022
9	River basin Shakhimardan (Archamazar gorge;		39.937579	71.754477	September 21–22, 2020
	Xirmandjay ridge;		39.925735	71.778222	May 28, 2022
	Mashalang (uzbek);		39.952897	71.752001	May 29, 2022
	Turbaza gorge;		39.966408	71.799734	May 30, 2021
	Koksu river		39.967476	71.850454	May 31, 2022
	Communication station;		39.952897	71.752001	August 6–7, 2021
	Iordan village)		39.942118	71.754948	August 8–10, 2021
10	Chap hills		40.939053	70.992813	May 18, 2021
11	The village of Gayrat		41.129928	71.956324	May 21, 2021
12	Yazyavan nature monument		40.607395 40.712742 40.724023	71.527129 71.468605 71.484	May 26, 2021 August 2, 2021 May 22, 2022
13	Ulugnar district road		40.804322	71.66175	May 27, 2021
14	Arbagish hill		41.259918 41.251027	71.896324 71.892680	June 17, 2021 June 13, 2022
15	Vorukh village road		40.339178	70.607855	June 1, 2021
16	Shirmanbulak village		40.579082	72.477385	July 21, 2021
17	Khanabad hills		40.823631	73.071675	July 22, 2021
18	Chartaksay		41.426868	71.677921	July 24, 2021
19	Kasansay district road		41.209391	71.466649	July 25, 2021
20	Kayrakkum desert		40.437343 40.434483	70.390235 70.403659	July 27, 2021 July 16, 2022
21	Shorsuv hills		40.238672 40.253238	70.822405 70.811175	July 28, 2021 June 20, 2022
22	Yazyavan desert		40.607462	71.499219	August 3, 2021
23	Kamchik pass		41.142028	70.446545	August 4, 2021
24	Almas village		41.061733	71.070558	May 23, 2022
25	Kasansay forestry		41.277463	71.526151	June 6, 2022
26	Khazratshah village		41.419472	71.764754	June 14, 2022
27	Lake Rezaksai		40.940997	71.331763	June 15, 2022
28	Lake Varzik		41.124029	71.270998	June 16, 2022
29	Narin river		41.014521	71.943849	July 11, 2022
30	Palvantash hills		40.597228	72.216148	July 13, 2022
31	Marhamat (Saritash hills)		40.458901	72.363365	July 14, 2022
32	Naukat village		40.515968	70.53903	July 17, 2022

**TABLE A2:** Websites that were consulted to collect more information about the recorded plants.

Database Name	Link
Global Biodiversity Information Facility (GBIF)	http://www.gbif.org/occurence
Global Plant Science (JSTOR)	http://plants.jstore.org
International Plant Name Index (IPNI)	http://www.ipni.org
IUCN	http://www.iucnredlist.org/details/
Kew world checklist of different plant families	http://wcsp.science.kew.org
Plantarium.ru	https://www.plantarium.ru
The Plant List	http://www.theplantlist.org
iNaturalist	https://www.inaturalist.org

**TABLE A3:** List of genera by number of taxa (*n* ≥ 5) in the tree flora of Fergana Valley.

Genus	Family	Number of species	Relative number (%)
*Rosa*	Rosaceae	11	6.66
*Prunus*	Rosaceae	10	6.06
*Salix*	Salicaceae	10	6.06
*Acantholimon*	Plumbaginaceae	10	6.06
*Astragalus*	Fabaceae	9	5.45
*Tamarix*	Tamaricaceae	8	4.84
*Lonicera*	Caprifoliaceae	8	4.84
*Calligonum*	Polygonaceae	6	3.63
*Crataegus*	Rosaceae	6	3.63
*Cotoneaster*	Rosaceae	5	3.03
*Atraphaxis*	Polygonaceae	5	3.03
11	Total	88	53.29

**TABLE A4:** Distribution of dendroflora species of the Fergana Valley according to the botanical‐geographical zoning scheme of Uzbekistan.

No.	Botanical‐geographical district	Species number	Botanical‐geographical regions	Species number
I Mountain Central Asian Province				
Tian–Shan mountain range				
1	I‐1 Western Tian–Shan	109	I‐1‐c Arashan	28
			I‐1‐d Kurama	15
			I‐1‐e Charkesar	103
2	I‐2 Fergana	58	I‐2‐a South–Chatkal	58
Pamir–Alay mountain range				
3	I‐3 Fergana–Alay	106	I‐3‐a Western–Alay	54
			I‐3‐b Eastern–Alay	87
II Turan Province				
4	II‐1 Central–Fergana	53	II‐1‐a Kayrakum–Yazyavan	45
			II‐1‐b East–Fergana	19
